# Dementia in Down’s syndrome: an MRI comparison with Alzheimer’s disease in the general population

**DOI:** 10.1186/1866-1955-5-19

**Published:** 2013-08-20

**Authors:** Diane Mullins, Eileen Daly, Andrew Simmons, Felix Beacher, Catherine ML Foy, Simon Lovestone, Brian Hallahan, Kieran C Murphy, Declan G Murphy

**Affiliations:** 1 Department of Forensic and Neurodevelopmental Sciences, Section of Brain Maturation, Institute of Psychiatry, De Crespigny Park, London, England, UK; 2 Department of Psychiatry, Royal College of Surgeons in Ireland, Education and Research Centre, Beaumont Hospital, Dublin 9, Ireland; 3 Department of Neuroimaging, Institute of Psychiatry, King’s College London, London, England, UK; 4 NIHR Biomedical Research Centre for Medical Health at the South London and Maudsley NHS Foundation Trust and King’s College London, Institute of Psychiatry, London, England, UK; 5 Section of Old Age Psychiatry, Institute of Psychiatry, De Crespigny Park, London, England, UK; 6 MRC Centre for Neurodegeneration Research, Section of Old Age Psychiatry, Institute of Psychiatry, King’s College London, London, England, UK; 7 Department of Psychiatry, Clinical Science Institute, National University of Ireland Galway, Galway, Ireland

**Keywords:** Dementia, Imaging, Intellectual disability

## Abstract

**Background:**

Down’s syndrome (DS) is the most common genetic cause of intellectual disability. People with DS are at an increased risk of Alzheimer’s disease (AD) compared to the general population. Neuroimaging studies of AD have focused on medial temporal structures; however, to our knowledge, no *in vivo* case–control study exists comparing the anatomy of dementia in DS to people with AD in the general population. We therefore compared the *in vivo* brain anatomy of people with DS and dementia (DS+) to those with AD in the general population.

**Method:**

Using MRI in 192 adults, we compared the volume of whole brain matter, lateral ventricles, temporal lobes and hippocampus in DS subjects with and without dementia (DS+, DS-), to each other and to three non-DS groups. These included one group of individuals with AD and two groups of controls (each age-matched for their respective DS and general population AD cohorts).

**Results:**

AD and DS+ subjects showed significant reductions in the volume of the whole brain, hippocampus and temporal lobes and a significant elevation in the volume of the lateral ventricle, compared to their non-demented counterparts. People with DS+ had a smaller reduction in temporal lobe volume compared to individuals with AD.

**Conclusions:**

DS+ and AD subjects have a significant reduction in volume of the same brain regions. We found preliminary evidence that DS individuals may be more sensitive to tissue loss than others and have less ‘cognitive reserve’.

## Background

Down’s syndrome (DS) is associated with trisomy of chromosome 21 and occurs in approximately 1 per 1,000 live births. It is the most common genetic cause of intellectual disability. People with DS encounter an additional disease burden because they have a significantly increased risk for developing Alzheimer’s disease (AD) in later life. In the general population, approximately 10% of 65-year-olds and 40% of 80-year-olds, develop symptoms of AD [1]. In contrast, the incidence of AD in people with DS is estimated to be three to five times higher. For example, at autopsy, the presence of Alzheimer-type neuritic plaques and neurofibrillary tangles have been reported in the brains of 7.5% of people with DS as early as the second decade of life, with a rise in prevalence to 80% of cases by the fourth decade and to 100% in individuals over 60 years of age [2].

The markedly high frequency of AD neuropathology and early onset of dementia in DS is poorly understood. Likely explanations, however, include a genetically determined elevation in risk factors for AD, and/or having less cognitive reserve due to a combination of these vulnerability factors and a pre-existing intellectual disability (mental retardation). For example, a significant proportion of the increased genetic risk for individuals with DS to develop dementia is probably explained by having trisomy of genes carried on chromosome 21 that are implicated in AD. Hence, it has been hypothesised that the presence of an extra copy of the amyloid precursor protein (APP) gene in individuals with DS leads to increased formation of amyloid plaques, neuronal death and clinical AD [3,4]. Similarly, trisomy of the myo-inositol (mI) transporter protein [5] is associated with an increase in brain mI, a compound that modulates neuronal development and survival, cellular osmolarity, membrane metabolism, signal transduction, protein C activation [6] and amyloid deposition [7]. We have previously reported [5] that subjects with DS who do not have dementia (DS-) have a significant increase in the concentration of mI as compared to controls, and that increased mI is associated with reduced overall cognitive ability (including memory). People with DS and dementia (DS+) have higher mI concentration than those without dementia (DS-), or people from the general population who have AD or mild cognitive impairment [8,9].

These DS-specific vulnerability factors may also combine with the additional burden of having a lower cognitive reserve due to pre-existing intellectual disability. The concept of brain reserve refers to the ability of the brain to tolerate the pathology of age- and disease-related changes without obvious clinical evidence [10]. The greater the reserve, the more severe pathological changes are needed to cause clinically functional impairment [10-12]. The cognitive reserve model suggests that the brain actively attempts to cope with brain damage by using pre-existing cognitive processing approaches or by enlisting compensatory ones [11].

Greater cognitive reserve can arise through numerous mechanisms, but is generally greater in people with higher overall intelligence, and/or those able to more efficiently/flexibly use brain networks [13]. Dementia risk has repeatedly been reported to be much lower in high-reserve individuals, but much higher in people with limited education and/or intellectual disability - a finding replicated across more than 20 studies involving more than 29,000 individuals and over a median follow-up period of greater than seven years [14]. Hence, it may be that dementia in people with DS is associated with less loss of brain tissue than in the general population because they have less cognitive reserve, due perhaps to a *double hit* of pre-existing intellectual disability combined with a genetically determined increase in risk factors such as brain amyloid and mI concentration.

In the general population, AD is associated with a loss of brain tissue, including from the temporal lobe and hippocampus [15-24], and the expansion of cerebrospinal fluid (CSF) [17,22,25-31]. Furthermore, AD severity is associated with progressive brain atrophy [32]. In comparison, there are relatively few magnetic resonance imaging (MRI) studies of dementia in DS. Nevertheless those that are available have reported that compared to DS-, DS+ have a significant reduction in the volume of the medial temporal lobe/hippocampus [33-35], in addition to a significant reduction in the volume of the whole brain and/or an increase in the volume of CSF [33,34,36].

To our knowledge, no *in vivo* case–control study has yet compared the anatomy of dementia in DS to people with AD in the general population. Therefore, we compared the volume of whole brain matter, lateral ventricles, temporal lobes and hippocampus in DS+ and DS- to each other, and to three non-DS groups. These included one group of individuals with AD and two groups of controls (each age-matched for their respective DS and general population AD cohorts). We tested the hypotheses that: (1) people with dementia (in both DS and in the general population) have a significant reduction in the volume of whole brain, temporal lobe and hippocampus and an increase in the volume of the lateral ventricles compared with controls, and (2) people with dementia and DS have less brain atrophy than people with AD in the general population.

## Method

### Participants

We included a total of 192 adults with successful MRI brain scans: 64 individuals with DS (19 DS+ and 45 DS-) and 128 adults without DS (43 younger healthy control subjects age-appropriate to the DS sample; and 46 older people with AD, together with 39 older healthy control subjects age-appropriate to the AD sample).

Individuals with genetically confirmed DS were recruited from community centres, residential homes and speciality clinics in London, Birmingham, Plymouth and Newcastle upon Tyne, UK. DS status was assessed in all participants by karyotyping and cognitive status was measured using the Cambridge Cognitive Examination (CAMCOG), a composite index of episodic memory, orientation, language, attention, praxis and executive function previously validated for use in DS [37]. The CAMCOG is appropriate for assessing cognitive function in people with intellectual disability, unlike more standard tests of cognitive function such as the Wechsler Adult Intelligence Scales. The CAMCOG incorporates, and is highly correlated with, the Mini Mental State Examination (MMSE) [38]. The decline in function of DS+ was based on the International Classification of Diseases-10 (ICD-10) Research Diagnostic Criteria. The AD samples were part of a larger, national longitudinal study based at the Institute of Psychiatry, London. Individuals from this study were diagnosed with dementia using the ICD-10 Research Diagnostic Criteria, with non-AD dementia excluded, in keeping with the National Institute of Neurological and Communicative Disorders and Stroke and Alzheimer’s Disease and Related Disorders Association (NINCDS-ADRDA) Criteria [14]. Age-appropriate, healthy controls (HC) were recruited from general practice lists and the local population as part of both studies. Absence of dementia was confirmed by screening with the CAMCOG and the MMSE.

All participants underwent standard physical, neurological and psychiatric screening, including routine clinical blood tests (for example, renal, liver and thyroid function). In addition, all participants underwent clinical MRI to exclude other brain disorders, including stroke or vascular dementia. Exclusion criteria included the presence of detectable physical disorder (for example, history of birth trauma or head injury), psychiatric illness (for example, major depression or psychosis) or other reason for cognitive decline (for example, changes in living situation). None of the participants were taking antipsychotic or antidepressant medication at the time of the study. However, seven DS+ (37%) and 25 AD (54%) participants were taking acetylcholinesterase (AChE) inhibitors.

It should be noted that we had a very high success rate in MRI scanning in our DS+ and AD groups, with less than 20% dropout/non-compliance across all recruited participants with dementia. There were no significant differences in age, gender, number of years in education or cognitive status between the dropouts and those from whom it was possible to obtain a successful scan. The study was approved by local and national Ethics Committees. After a complete description of the study was provided to the participant and the identified carer, written informed consent was obtained where possible. Where not possible, the participant’s assent was obtained with formal consent provided by an identified carer.

### MRI protocol

Subjects were scanned using a 1.5 Tesla, GE NV/i Signa MR system at the Maudsley Hospital, London. A vacuum fixation device ensured that subjects were both comfortable and restrained from movement during the scanning process. The whole brain was imaged with three-dimensional inversion recovery prepared fast spoiled gradient-recalled acquisition in the steady state (SPGR) T1-weighted dataset. These T1-weighted images were obtained in the axial plane with 1.5-mm contiguous sections, repetition time (TR) of 13.8 milliseconds, inversion time (TI) of 450 milliseconds, echo time (TE) of 2.8 milliseconds and flip angle of 20˚ with one data average and a 256 × 256 × 124 matrix. Image contrast for all datasets was chosen with the aid of optimising software [39]. Acquisition time was 6 minutes, 27 seconds. Full brain and skull coverage was required and a detailed quality control was carried out on all MR images according to previously described quality control procedures [40,41].

### MRI data analysis

Volumetric analysis of hippocampi, temporal lobes, lateral ventricles, whole brain matter and total intracranial volumes (TIV) were performed. Manual tracing was performed on SPGR data sets using both Measure Image Analysis Software [42,43] (Johns Hopkins University, Baltimore, Maryland, USA) and published anatomical definitions [43,44]. Whole brain matter volume consisted of total cerebral hemispheres (frontal, temporal, parietal and occipital lobes) and excluded lateral and third ventricles. The three-dimensional acquisition as utilised, well differentiated brain parenchyma from CSF (including peripheral CSF, lateral and third ventricles) and was based upon differences in pixel intensities in signal as derived from the T1 weighted sequences employed. Intracranial volume included all brain matter (cerebral hemispheres, cerebellum and brainstem) lateral and third ventricles and all peripheral CSF in the cranium. The extra axial boundaries were derived from analysis of the signal of the dura and diploic space, permitting identification of the subdural contents. At the base of the brain, the dural signal line was carefully followed and where there was any interruption in this signal, raters utilised their knowledge of neuroanatomy to estimate the line best connecting the dural signal. The temporal lobes included all pixels traced from the anterior pole of the temporal lobe to the aqueduct of Sylvius with the superior temporal lobe boundary defined as a straight line drawn from the angle of the medial temporal lobe, where it was attached to the temporal stem, to the midpoint of the operculum. The dura of the middle cranial fossa was then traced around each temporal lobe to complete the temporal lobe region [44]. The hippocampus was traced by means of an adaptation of the criteria of Watson *et al*. [45] - namely, we did not trace the hippocampus any further than the aqueduct of Sylvius. The superior and inferior horns of the lateral ventricles were measured from their first appearance in the frontal and temporal lobes, respectively, terminating at the atrium. The posterior horn of the lateral ventricles was measured from the atrium to its last appearance (on the coronal slice) in the occipital lobe. Raters were blind to subject status. The volume of each region was calculated by multiplying the summed pixel cross-sectional areas by slice thickness. Intra-rater and inter-rater reliabilities were determined for the brain regions of interest (ROIs) traced by the operators as part of this analysis. Inter-rater reliabilities were obtained for all regions traced [46]. The intra-rater and inter-rater reliabilities were intraclass correlation coefficients. For all regions r was >0.9 for the inter-and intra-rater correlation coefficients.

In order to control for the relationship between brain volume and head size, volumes were expressed as raw (uncorrected) volumes, and when normalised, as a percentage of traced TIV. Statistical analyses were carried out on both raw and corrected brain volumes. TIV is determined during childhood by the volume of brain, meninges, and CSF contained within it [47,48]. Brain volume is maximal by early childhood and appears to decline from early adulthood. The normalisation to TIV provided the proportion of hippocampal volume to past brain size.

### Statistical analysis

Subject groups were normally distributed. All volumes were normally distributed and were analysed using univariate analysis. However, age was significantly different between groups (F 157.556, *P* <0.001), as was the gender distribution. Age, gender and TIV were added as covariates in the analysis. CAMCOG scores were corrected for age. Follow-up pairwise comparisons among estimated marginal means, adjusting for covariates, were conducted where appropriate. Pairwise analyses were adjusted for multiple tests. All significance tests used a *P*-value of <0.05 adjusted for multiple testing using the Bonferroni correction. The percentage reduction in the volume of the raw and TIV-corrected volumes of the hippocampus and temporal lobe (and the percentage increase in the volume of the lateral ventricles) within individuals with DS between DS+ and DS-, were compared to those within AD cases and controls from the general population by means of the *t*-test. The percentage differences were based on age-corrected volumes. Our results may have been confounded by medication status, therefore, we compared brain anatomy in individuals with dementia who were taking AChE inhibitors and those who were not taking such medication.

## Results (Table 1)

**Table 1:** Magnetic resonance imaging comparing subjects with Down’s syndrome and Alzheimer’s disease in the general population

	**DS+ (n = 19) mean ± SD**	**DS- (n = 45) mean ± SD**	**DS HC (n = 43) mean ± SD**	**AD (n = 46) mean ± SD**	**AD HC mean ± SD**	**F effect of group (*P*-value)**	**F effect of gender (*P*-value)**	**Significant pairwise comparisons**
Age (years)^*^	51.52 ± 7.89	38.07 ± 12.24	33.75 ± 11.37	76.59 ± 5.3	75.87 ± 5.53	157.556 (<0.001)	NS	DS+<AD; DS+<AD HC; DS-<AD; DS-<DS+; DS HC<AD; DS HC<DS+; DS HC<AD HC
Education (years)				11.13 ± 3.22	11.49 ± 3	NS	NS	NS
Sex (female:male)	10:9	31:14	29:14	22:24	11:28			
MMSE^*^	9.32 ± 4.46	13.88 ± 5.56	15.23 ± 2.53	22.48 ± 3.74	28.74 ± 3.23	35.757 (<0.001)	NS	AD<AD HC; DS+<AD; DS+<AD HC; DS-<AD; DS-<AD HC; DS HC<AD; DS HC<AD HC
CAMCOG^*^	33.72 ± 19.77	52.98 ± 21.48	114.83 ± 16.52			59.323 (<0.001)	NS	DS+<DS HC; DS-<DS HC
Whole brain volume (WBV, ml)^*^	836.33 ± 98.72	961.96 ± 111.16	1100 ± 101.23	904.32 ± 83.3	930.77 ± 77.41	23.296 (<0.001)	28.717 (<0.001)	DS+<AD; DS+<AD HC; DS+<DS HC; DS-<AD HC; DS-<DS HC; DS+<DS-
Total intracranial volume (TIV, ml)^*^	1096.46 ± 97.76	1195.7 ± 120.08	1387.95 ± 125.08	1292.39 ± 109.27	1277.27 ± 97.95	38.112 (<0.001)	55.296 (<0.001)	DS+<AD; DS+<AD HC; DS+<DS HC; DS-<AD; DS-<AD HC; DS-<DS HC
Hippocampus (ml)^*^	4.52 ± 1.06	5.56 ± 0.81	6.82 ± 0.62	5.13 ± 1.04	6.19 ± 0.85	13.242 (<0.001)	NS	AD<AD HC; AD<DS HC; DS+<DS-; DS+<AD HC; DS+<DS HC; DS-<DS HC
Hippocampal volume normalised by TIV (% TIV)^*^	0.41 ± 0.09	0.47 ± 0.07	0.49 ± 0.05	0.4 ± 0.07	0.5 ± 0.06	13.095 (<0..001)	NS	AD<DS HC; AD<AD HC; DS+<DS-; DS+<AD HC; DS+<DS HC
Left hippocampus (ml)^*^	2.37 ± 0.58	2.97 ± 0.59	3.52 ± 0.38	2.62 ± 0.51	3.17 ± 0.46	12.723 (<0.001)	NS	AD<AD HC; AD<DS HC; DS+<DS HC; DS-<DS HC
Left hippocampus normalised by TIV (% TIV)^*^	0.22 ± 0.05	0.24 ± 0.03	0.25 ± 0.03	0.2 ± 0.03	0.25 ± 0.03	12.339 (<0.001)	NS	AD<DS-; AD<AD HC; AD<DS HC; DS+<DS HC; DS+<DS-
Right hippocampus (ml)^**^	2.16 ± 0.52	2.7 ± 0.47	3.31 ± 0.31	2.48 ± 0.58	3.02 ± 0.44	11.540 (<0.001)	NS	AD<AD HC; AD<DS HC; DS+<DS HC; DS+<AD HC; DS+<DS-
Right hippocampus normalised by TIV (% TIV)^*^	0.2 ± 0.04	0.23 ± 0.04	0.24 ± 0.03	0.19 ± 0.04	0.24 ± 0.03	11.660 (<0.001)	NS	AD<AD HC; AD<DS HC; DS+<AD HC; DS+<DS HC; DS+<DS-
Temporal lobes (ml)^*^	104.23 ± 16.84	121.75 ± 14.95	136.16 ± 16.65	101.78 ± 15	110.57 ± 14.21	5.947 (<0.001)	NS	AD<DS-; AD<AD HC
Temporal lobes normalised by TIV (% TIV)^*^	9.48 ± 1.1	10.2 ± 0.93	9.8 ± 0.77	7.86 ± 0.81	8.65 ± 0.8	5.998 (<0.001)	4.2 (0.042)	AD<DS-; AD<AD HC
Left temporal lobe (ml)^*^	52.05 ± 8.55	61.26 ± 7.68	68.72 ± 8.15	51.97 ± 8.35	56.74 ± 9.61	4.967 (0.001)	NS	AD<AD HC; AD<DS-
Left temporal lobe normalised by TIV (% TIV)^**^	4.73 ± 0.57	5.12 ± 0.48	4.95 ± 0.4	4.02 ± 0.51	4.43 ± 0.58	4.851 (0.001)	NS	AD<AD HC; AD<DS-
Right temporal lobe (ml)^**^	52.17 ± 8.7	60.02 ± 7.81	67.37 ± 9.63	49.8 ± 9.14	53.82 ± 7.21	3.067 (0.018)	NS	AD<AD HC
Right temporal lobe normalised by TIV (% TIV)^**^	4.74 ± 0.54	5.0 ± 0.57	4.85 ± 0.49	3.84 ± 0.57	4.22 ± 0.51	3.112 (0.017)	NS	AD<AD HC
Lateral ventricles (ml)^*^	26.78 ± 21.32	17.21 ± 15.94	10.56 ± 8.38	49 ± 21.81	27.72 ± 12.27	9.238 (<0.001)	NS	AD>AD HC; AD>DS HC
Lateral ventricles normalised by TIV (% TIV)^*^	2.44 ± 1.86	1.43 ± 1.24	0.76 ± 0.61	3.8 ± 1.65	2.16 ± 0.89	9.009 (<0.001)	NS	AD>AD HC; AD>DS HC
Left lateral ventricle (ml)^*^	12.7 ± 12.08	7.52 ± 6.47	5.26 ± 4.36	22.68 ± 10.75	13.6 ± 6.07	7.115 (<0.001)	NS	AD>AD HC; AD>DS HC
Left lateral ventricle normalised by TIV (% TIV)^*^	1.16 ± 1.04	0.63 ± 0.51	0.38 ± 0.32	1.76 ± 0.82	1.06 ± 0.45	6.967 (<0.001)	NS	AD>AD HC; AD>DS HC
Right lateral ventricle (ml)^**^	14 ± 9.69	9.66 ± 9.58	5.3 ± 4.36	26.17 ± 11.63	14.11 ± 6.7	10.3 (<0.001)	NS	AD>AD HC; AD>DS HC; DS+>DS HC
Right lateral ventricle normalised by TIV (% TIV)^**^	1.27 ± 0.86	0.8 ± 0.7	0.38 ± 0.31	2.0 ± 0.88	1.1 ± 0.48	9.995 (<0.001)	NS	AD>AD HC; AD>DS HC; DS+>DS HC

^*^P <0.001; ^**^P <0.05. Age, gender and TIV were added as covariates in the analysis. DS+, people with Down’s syndrome and dementia; DS-, people with Down’s syndrome without dementia; *DS HC* Down’s syndrome healthy controls, *AD* Alzheimer’s disease, *AD HC* Alzheimer’s disease healthy controls, *MMSE* Mini Mental State Examination, *CAMCOG* Cambridge cognitive examination, *TIV* total intracranial volume, *NS* not significant.

### Raw (uncorrected) volumes

There was a significant main effect of group for the hippocampus, temporal lobes and lateral ventricles. There was a significant main effect of group and gender for whole brain volume (WBV) and TIV. We found no significant differences between DS+ who were taking AChE inhibitors and those who were not.

**Figure 1:**
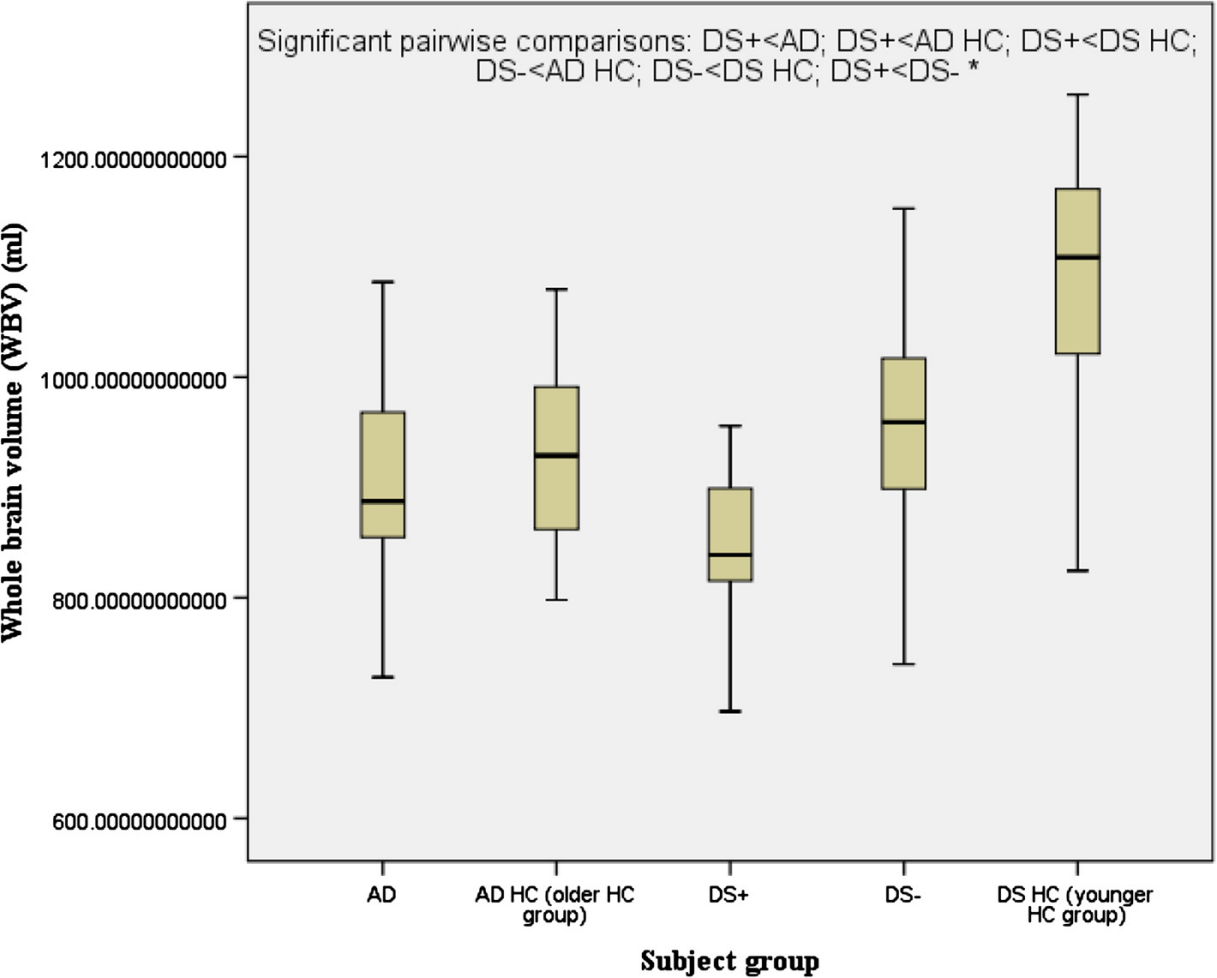
**Whole brain volume.** Comparison of people with Alzheimer’s disease (AD) versus those with Down’s syndrome and dementia (DS+). HC, healthy controls; DS-, people with Down’s syndrome without dementia. ^*^P <0.0001.

Follow-up pairwise comparisons revealed that compared to their respective control groups without dementia, both DS+ and AD had a highly significant reduction in the volume of the hippocampus, and AD (but not DS+) had a highly significant reduction in the volume of the temporal lobe and a highly significant increase in the volume of the lateral ventricles. DS+ had a highly significant reduction in the volume of the hippocampus compared to DS-. Compared to the DS healthy control group (the younger healthy control group), both DS+ and DS- had a highly significant reduction in WBV (Figure 1). The hippocampal and temporal lobe volume reductions in AD and DS+ were disproportionately greater than the WBV reduction.

The percentage differences between DS+ and DS-, were compared to those within AD cases and controls from the general population and were found to be significant. The reduction in the volume of the hippocampus between DS+ and DS- was similar to that within AD cases and controls from the general population (19% and 17%, respectively). In contrast, the reduction in the volume of the temporal lobe between DS+ and DS- was almost twice that within AD cases and controls from the general population (14% and 8%, respectively). The increase in volume of the lateral ventricles between DS+ and DS- was less than that within AD cases and controls from the general population (36% and 43% respectively).

### Volumes corrected for TIV

**Figure 2:**
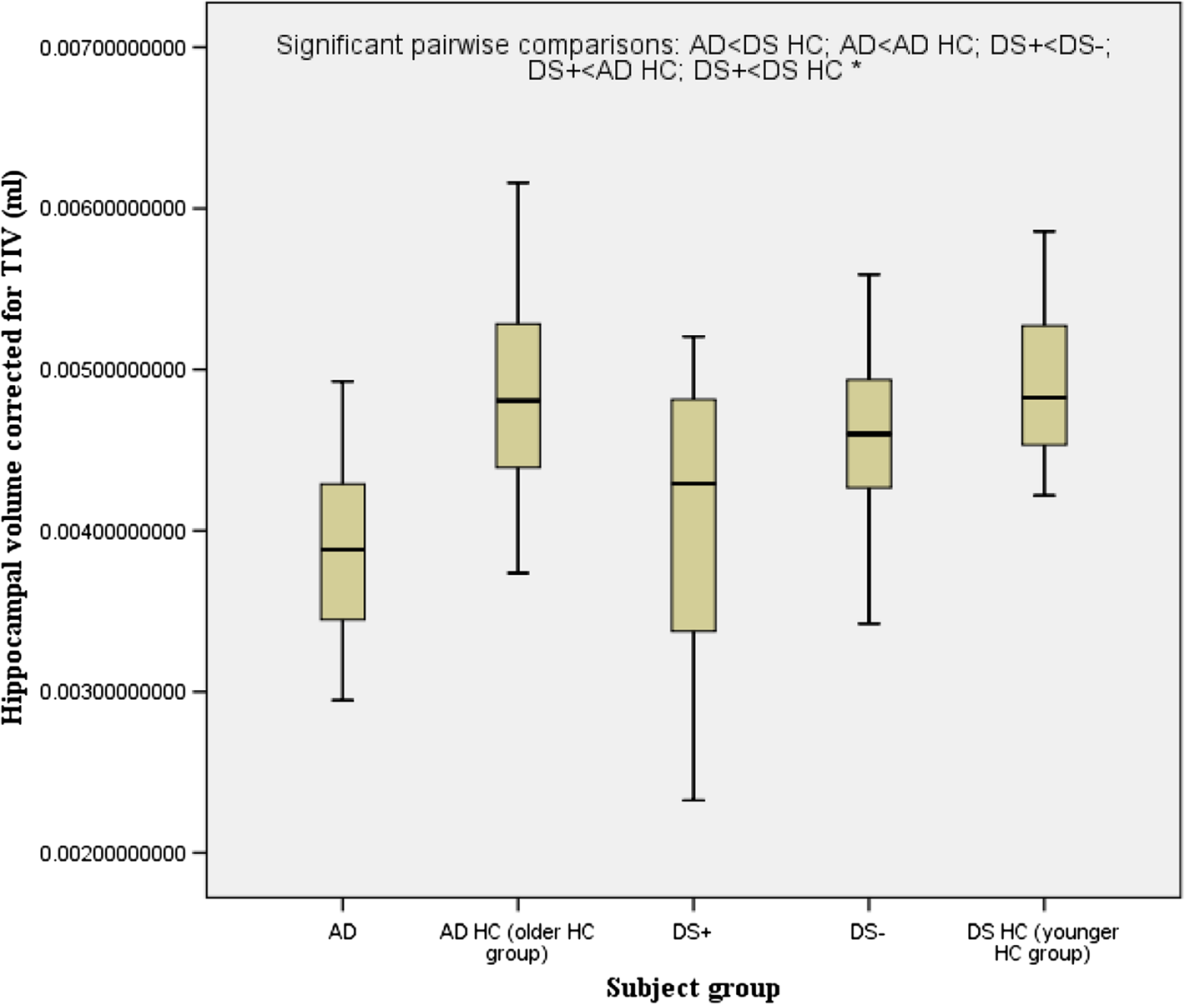
**Hippocampal volume corrected for total intracranial volume (TIV) (corrected as a ratio of the hippocampal volume to TIV).** Comparison of people with Alzheimer’s disease (AD) and people with Down’s syndrome with dementia (DS+). HC, healthy controls; DS-, people with Down’s syndrome without dementia. ^*^P <0.0001.

**Figure 3:**
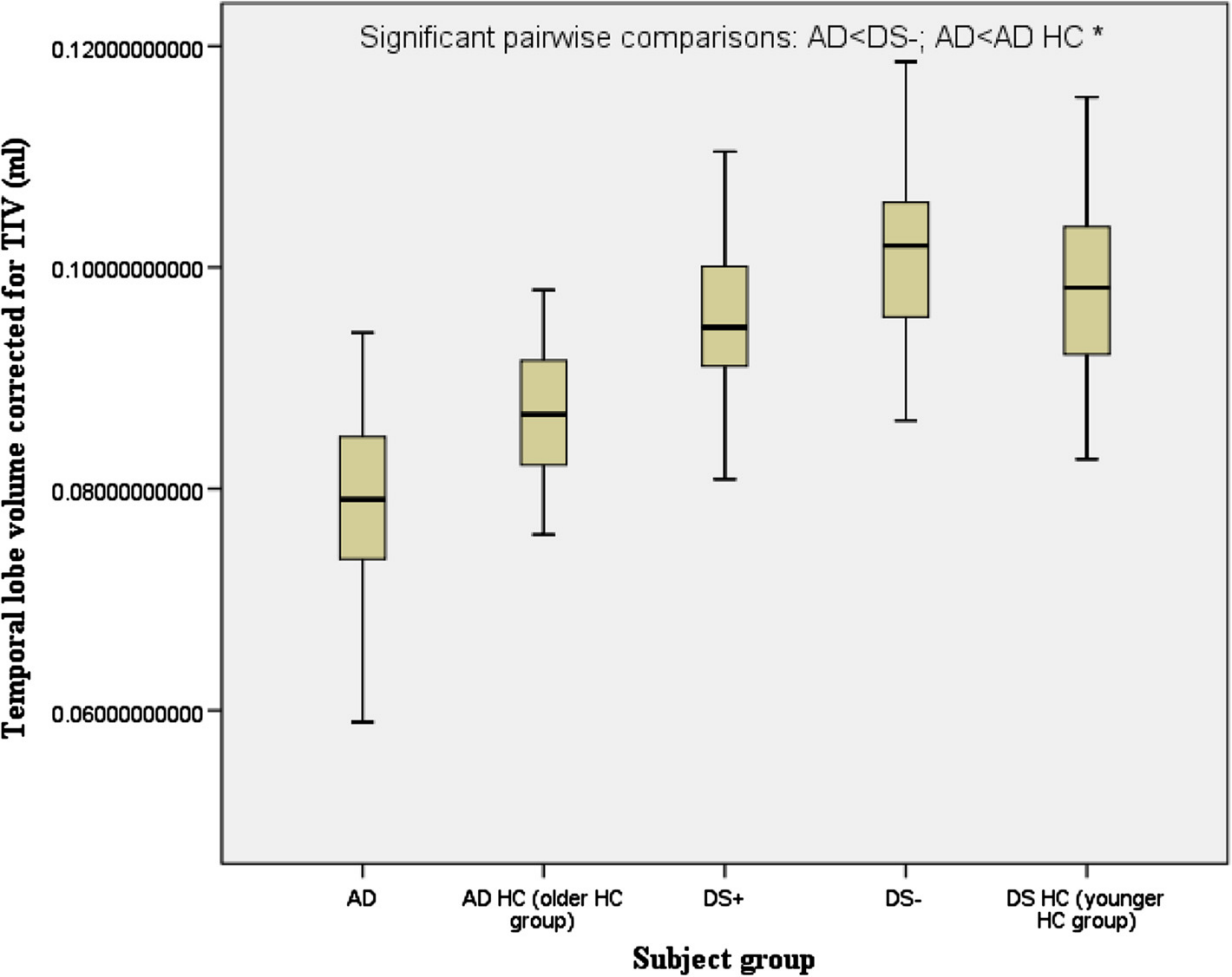
**Temporal lobe volume corrected for total intracranial volume (TIV) (corrected as a ratio of the temporal lobe volume to TIV).** Comparison of people with Alzheimer’s disease (AD) and people with Down’s syndrome with dementia (DS+). HC, healthy controls; DS-, people with Down’s syndrome without dementia. ^*^P <0.0001.

**Figure 4:**
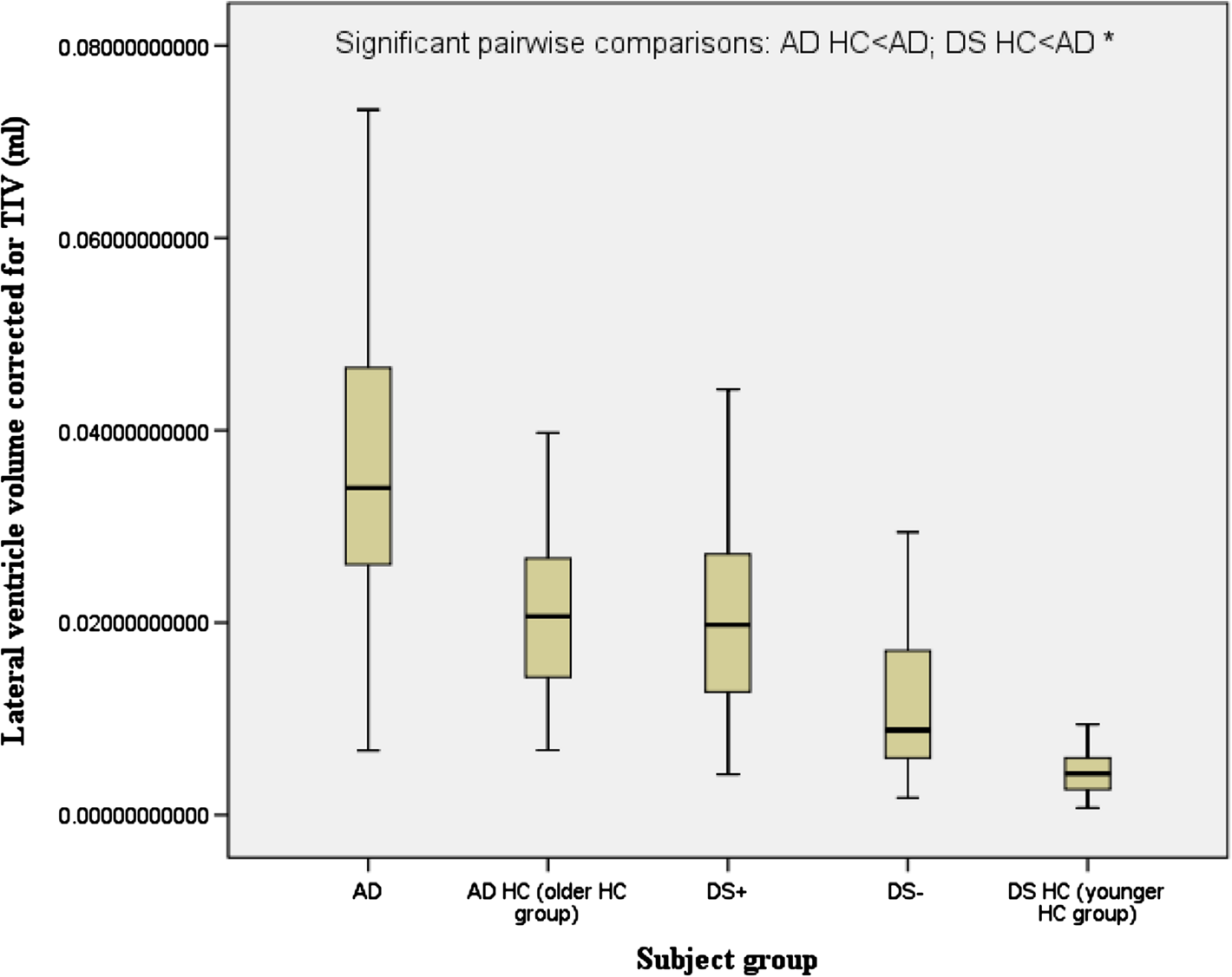
**Lateral ventricle volume corrected for total intracranial volume (TIV) (corrected as a ratio of the lateral ventricle volume to TIV).** Comparison of people with Alzheimer’s disease (AD) and people with Down’s syndrome with dementia (DS+). HC, healthy controls; DS-, people with Down’s syndrome without dementia. ^*^P <0.0001.

There was a significant main effect of group for the hippocampus, temporal lobes and the lateral ventricles. There was a significant main effect of gender for the temporal lobe. Follow-up pairwise comparisons revealed that compared to their respective control groups without dementia, both DS+ and AD had a highly significant reduction in the volume of the hippocampus (Figure 2), and AD (but not DS+) had a highly significant reduction in the volume of the temporal lobe (Figure 3) and a highly significant increase in the volume of the lateral ventricles (Figure 4). DS+ had a highly significant reduction in the volume of the hippocampus compared to DS-.

The percentage differences between DS+ and DS-, were compared to those within AD cases and controls from the general population and were found to be significant. The reduction in the volume of the hippocampus between DS+ and DS- was less than half that within AD cases and controls from the general population (7% and 15% respectively). Similarly, the reduction in the volume of the temporal lobe between DS+ and DS- was also less than half that within AD cases and controls from the general population (2% and 5%, respectively). The increase in volume of the lateral ventricles between DS+ and DS- was similar to that between AD and AD HC (41% and 40%, respectively).

### Relationship of cognitive ability to brain anatomy

In the AD population, there was a positive correlation between MMSE and corrected hippocampal volume (*r* = 0.311, *P* = 0.01) and between MMSE and corrected temporal lobe volume (*r* = 0.316, *P* = 0.05). There was a negative correlation between MMSE and the corrected lateral ventricle volume (*r* = − 0.475, *P* = 0.01). The DS+ population showed a positive correlation between CAMCOG and the corrected hippocampal volume (*r* = 0.216, *P* = 0.05) and between CAMCOG and the corrected temporal lobe volume (*r* = 0.435, *P* = 0.01). There was a negative correlation between MMSE and the corrected lateral ventricle volume (*r* = − 0.462, *P* = 0.01).

## Discussion

In this study, we found that people with dementia (both with DS and AD) had a reduction in the volume of whole brain, temporal lobe and hippocampus, in addition to an elevation in lateral ventricle volume, compared to controls. Also, for the first time, we found that DS+ had a relatively smaller reduction in brain volume compared to people with AD in the general population. Following normalisation and correction for confounders, it was found that both the AD and DS+ groups had a significant reduction in hippocampal volume when compared to their comparison control groups. The AD group also showed a significant reduction in temporal lobe volume and a significant increase in lateral ventricle volume compared to its age-matched control group.

Subjects with DS are at an increased risk for dementia, thought to be of the AD type [47-49]. The hippocampal volumes in DS+ in this study were disproportionably small compared to age-matched healthy controls. This is in agreement with previous neuropathological [44] and neuroimaging studies of subjects with DS [32,33,50-52] and a number of MRI studies of AD subjects in the general population [23,24,53-55]. Reduced hippocampal volume however, is not a feature of all people with an intellectual disability. In subjects with fragile X syndrome [56] and autism [57], for example, corrected hippocampal volume is reported to be significantly increased compared to healthy controls. Therefore, reduced hippocampal volume in the brains of subjects with DS does not appear to simply reflect a non-specific effect of an intellectual disability.

In individuals with DS, the reduction in the volume of the hippocampus in DS+ compared to DS- was less than half that in people with AD compared to controls from the general population (7% and 15%, respectively). Similarly, the reduction in the volume of the temporal lobe in DS+ compared to DS- was also less than half that in people with AD compared to controls from the general population (2% and 5%, respectively). The conclusion that DS+ subjects have less brain volume than AD subjects was therefore based on the TIV-corrected percentage differences between the hippocampus and temporal lobe volumes for DS+ versus DS-, as compared with AD versus AD HC. In this study DS- had a mean age of 38 years but did not show a significant difference from DS HC for the volumes of the lateral ventricle or for the lateral ventricle volume corrected for TIV. DS- in this study are very likely to develop dementia as they age.

In corroboration with our findings, a number of previous studies have demonstrated a reduction in the volume of the hippocampus in individuals with DS compared to healthy controls, utilising MRI [50,58,59]. Furthermore, DS- have also been shown to have smaller hippocampi compared to healthy controls [51], with an age-related reduction in hippocampal volume compared to controls also evident [60].

Post-mortem studies have reported that adults with DS have prominent neuropathology in the medial temporal lobe structures in the early stages of AD [61-64]. In the current study, the volumetric findings in subjects with DS were consistent with an AD pattern of atrophy, with a reduction in the volume of the hippocampus. This study tentatively supports the finding that the hippocampus is one of the brain regions most severely affected by amyloid plaques and neurofibrillary plaques in DS [63] and that a reduction in hippocampal volume may provide a useful tool to assist the diagnosis of dementia in people with DS, as has been proposed for people with AD in the general population [65,66].

The first volumetric MRI study of AD published in 1988 described a 40% reduction in the volume of the hippocampus of subjects with AD compared to HC [67]. Subsequent studies have similarly reported reduced volume of the hippocampal and parahippocampal formation in AD of 20% to 52% [68], with this volume reduction already present at the first stages of AD [69-71]. The results of the current study showed that in individuals with DS, the reduction in the volume of the hippocampus in DS+ compared to DS- was similar to that in people with AD compared to controls from the general population (19% and 17%, respectively). However, atrophy of the hippocampus is not specific to AD and occurs in other forms of dementia [72,73]. Despite this, hippocampal volume has been shown to be superior to clinical diagnosis or cognitive assessment in predicting AD neuropathology [74].

We demonstrated a reduction in temporal lobe volume in subjects with AD compared to their age-matched HC, a finding consistent with previous MRI volumetric studies [75-80]. The medial temporal lobe plays an important role in the storage of new information [81,82] and atrophy of the medial temporal lobe may explain why memory dysfunction is an early symptom of AD [83,84]. Consistent with this, subjects with memory impairment who do not meet the criteria for dementia have an increased risk of subsequent AD [85-87]. In the same way, atrophy of the hippocampus increases the risk for subsequent AD in elderly individuals without dementia [88,89] and for asymptomatic individuals at risk of autosomal dominant AD [90].

The cognitive-reserve hypothesis concerns the percentage of the brain that is occupied by the hippocampus. People with a greater proportion of their brains occupied by their hippocampus would therefore have an increased relative reserve. Individuals with higher levels of cognitive reserve will have a lower prevalence and incidence of dementia [91]. It is, therefore, a reduction in the volume of the hippocampus corrected by TIV (relative reserve) rather than a lack of raw hippocampal volume (reserve based on actual tissue volume) that makes one more susceptible to dementia. In the current study, follow-up pairwise comparisons revealed that compared to their respective control groups without dementia, both DS+ and people with AD had a highly significant reduction in the volume of the hippocampus, which had been corrected for TIV.

It has been hypothesised that in the brain of people with DS, the presence of an extra copy of the amyloid precursor gene located on chromosome 21 leads to abnormalities in APP-processing in neuronal membranes, and subsequently to amyloid plaques and DS [3] However, mI also promotes the formation of amyloid plaques [7]. The cause of the elevation in mI concentration in the brain in DS and how this affects cognitive ability is unknown [5]. It is possible that the increase in mI concentration does not directly affect hippocampal neuronal function but simply reflects another underlying metabolic process that is more closely linked with neuronal dysfunction [5]. Therefore, the predisposition of people with DS to amyloid deposition and subsequent AD may arise from a significantly higher gene dose of both the APP and the mI transport genes [5].

A higher level of cognitive functioning has been shown to be associated with fewer cases of dementia in individuals with DS, and the level of cognitive functioning appears to be associated with environmental factors such as the level of education, years in an institution and employment [92]. Furthermore, individuals with lower levels of intellectual functioning are expected to experience an earlier onset of dementia symptoms and a faster rate of decline [92]. In addition, people with DS may develop symptoms of AD earlier in life than other individuals because of their smaller cognitive reserve and their increased production of amyloid beta. Hence it may be that dementia in people with DS can occur with less loss of brain tissue than in the general population because they have less cognitive reserve due perhaps to a *double hit* of pre-existing intellectual disability combined with a genetically determined increase in risk factors such as brain amyloid and mI concentration.

### Relationship of cognitive ability to brain anatomy

The results of this study showed hippocampal and temporal lobe volume reductions in AD and DS+, with these volume reductions correlated with cognitive decline in both groups. A positive correlation was demonstrated in the AD group for MMSE score and for both corrected hippocampal volume and corrected temporal lobe volume. This finding is corroborated by previous research also demonstrating that performance on the MMSE was directly correlated with hippocampal volume [61]. The correlation between MMSE and the volume in these critical areas, suggests that the function of the hippocampus and temporal lobe is compromised when the volume is reduced. One may thus speculate that severe medial temporal lobe atrophy is associated with greater cognitive decline, a proposal in line with the finding that a small hippocampal volume at baseline is associated with a decrease in cognitive scores during follow up [93].

### Limitations

The initial findings that people with dementia (both with DS and AD) had a reduction in the volume of whole brain, temporal lobe and hippocampus; in addition to an elevation in lateral ventricle volume, compared to controls, were potentially confounded by significant between-group differences in brain size, age and gender. To overcome the potential confounder of brain size, all volumes were corrected for TIV.

Age was significantly different between the groups and is a confounder because age-related reductions in medial temporal brain regions, including the hippocampus, have previously been demonstrated in DS [32,58,94,95]. While all study groups ideally would be equivalent in age, including AD and DS+, this is virtually impossible in this study. Life expectancy estimates suggest that only 14% of the DS population (with dementia or otherwise) reach the age of 68 years [96]. Furthermore, the neuropathology of AD and some degree of the gross neuroanatomical change associated with AD have been reported to occur in the brains of 80% of individuals with DS by the fourth decade and of 100% over 60 years of age [2]. Therefore, attaining DS- and in particular DS+ groups over 65 years to match AD and control groups over 65 years of age, is not feasible. Thus, unlike some previous MRI studies [32,33] we co-varied for age. We also corrected for gender as there was a higher proportion of female subjects in the AD HC group, and because gender is associated with differential degrees of brain volume reduction, including hippocampal volume reduction, with aging [44].

Another possible confounder for our results is that some DS individuals were taking AChE inhibitors and others were not. The reasons for this discrepancy are unknown, but it most probably reflects differences in local prescribing habits. We cannot state whether or not medication status in our DS+ and AD sample was related to length of illness. This is because it is often difficult to accurately access the date of onset of dementia in people with DS, and we were not able to retrospectively establish the time of AD onset or the length of illness. We found no significant differences between those participants with dementia who were taking AChE inhibitors and those who were not. Nonetheless, it remains possible that medication status was a significant confounder for our analysis. However, many DS+ are treated with AChE inhibitors and, therefore, we decided not to exclude these participants.

This was a cross-sectional study and clinical rather than post-mortem criteria were used to identify subjects with AD and DS+. It is not possible to be certain that all of the people with dementia under investigation had AD, as this can only be definitively addressed at autopsy. Nevertheless, all the individuals with dementia from both groups (DS and AD in the general population) were diagnosed using standardised instruments and individuals with detectable cerebrovascular disease were excluded. Therefore, the group differences found in regional brain volume between DS+ and AD in the general population most probably reflect AD-type neuropathology. The sample in this study was characterized with AD using identical methods to those previously reported and accepted for publication by other high impact factor journals [5,9,36].

Our research laboratory has significant experience in the use of Measure software and our inter-rater and intra-rater correlation coefficients were >0.9. Due to our experience of this software, we chose to use it in this study. However, there are other packages, namely the semi-automated package, FreeSurfer, which can produce more detailed metrics, including information on cortical thickness.

## Conclusion

To our knowledge, this is the first study to compare the anatomy of DS+ with individuals with AD. While similar deficits existed in both DS+ and people with AD, namely, reduced volume of the hippocampus and temporal lobe regions compared to their respective control groups, it is possible that less significant regional brain volume reductions are required for the presence of dementia in people with DS. This is potentially explained by their lower cognitive reserve.

## Abbreviations

AChE: Acetylcholinesterase; AD: Alzheimer’s disease; APP: Amyloid precursor protein; CAMCOG: Cambridge cognitive examination; CSF: Cerebrospinal fluid; DS: Down’s syndrome; DS+: People with Down’s syndrome and dementia; DS-: People with Down’s syndrome without dementia; HC: Healthy controls; ICD-10: International classification of diseases-10; mI: Myo-inositol; MMSE: Mini mental state examination; MRI: Magnetic resonance imaging; NINCDS-ADRDA: National Institute of Neurological and Communicative Disorders and Stroke and Alzheimer’s Disease and Related Disorders Association; ROI: Region of interest; SPGR: steady state; TE: Echo time; TI: Inversion time; TR: Repetition time; TIV: Total intracranial volume; WBV: Whole brain volume.

## Competing interests

All authors declare that they have no competing interests.

## Authors’ contributions

DM undertook the analysis and interpretation of data, and drafting the article. DM, BH, KCM and DGM revised the article critically for important intellectual content. All authors were involved in the conception and design of the study, and the final approval of the version to be published.
